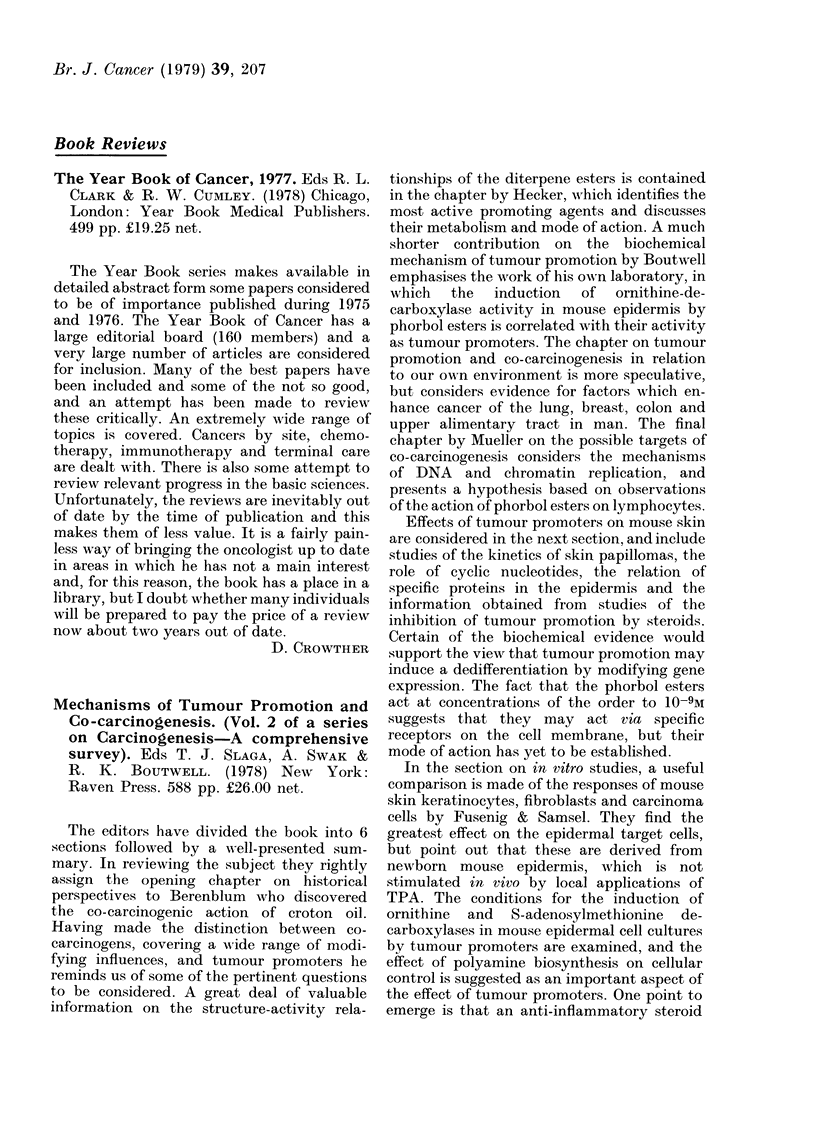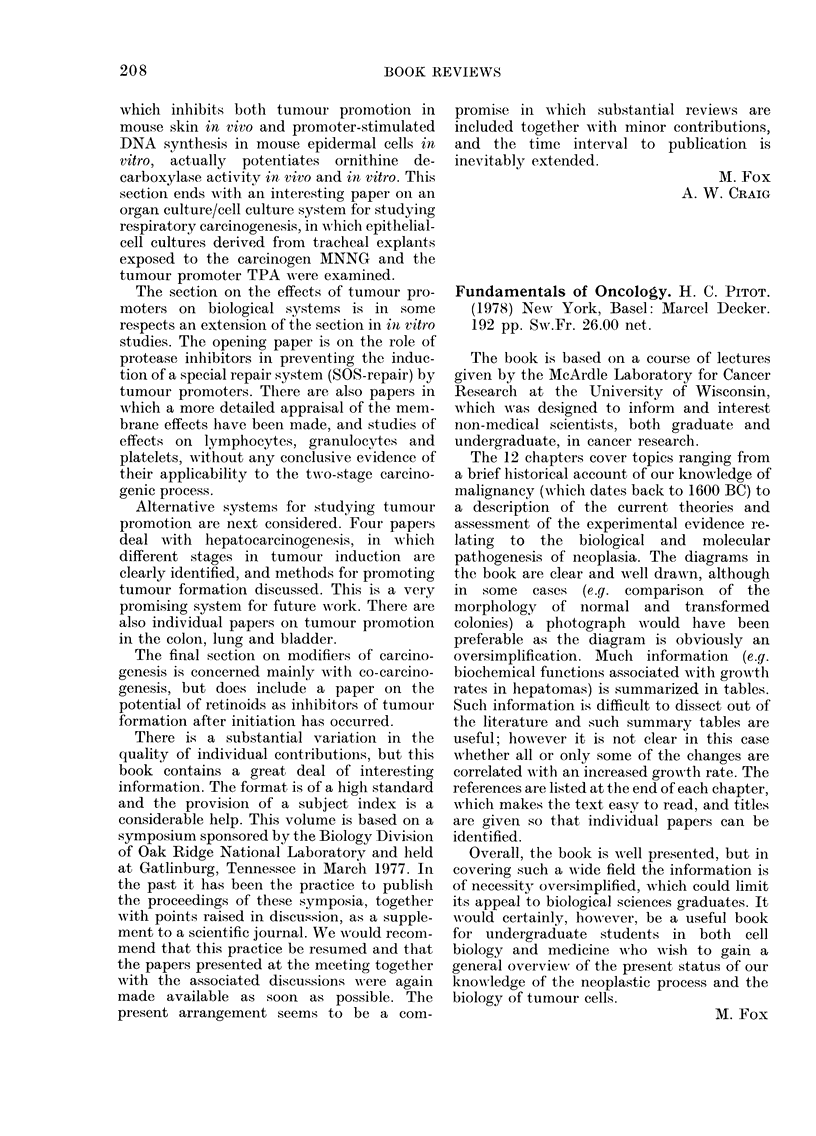# Mechanisms of Tumour Promotion and Co-carcinogenesis. (Vol. 2 of a series on Carcinogenesis—A comprehensive survey)

**Published:** 1979-02

**Authors:** M. Fox, A. W. Craig


**Mechanisms of Tumour Promotion and Co-carcinogenesis. (Vol. 2 of a series on Carcinogenesis—A comprehensive survey).** Eds T. J. Slaga, A. Swak & R. K. Boutwell. (1978) New York: Raven Press. 588 pp. £26.00 net.

The editors have divided the book into 6 sections followed by a well-presented summary. In reviewing the subject they rightly assign the opening chapter on historical perspectives to Berenblum who discovered the co-carcinogenic action of croton oil. Having made the distinction between co-carcinogens, covering a wide range of modifying influences, and tumour promoters he reminds us of some of the pertinent questions to be considered. A great deal of valuable information on the structure-activity relationships of the diterpene esters is contained in the chapter by Hecker, which identifies the most active promoting agents and discusses their metabolism and mode of action. A much shorter contribution on the biochemical mechanism of tumour promotion by Boutwell emphasises the work of his own laboratory, in which the induction of ornithine-decarboxylase activity in mouse epidermis by phorbol esters is correlated with their activity as tumour promoters. The chapter on tumour promotion and co-carcinogenesis in relation to our own environment is more speculative, but considers evidence for factors which enhance cancer of the lung, breast, colon and upper alimentary tract in man. The final chapter by Mueller on the possible targets of co-carcinogenesis considers the mechanisms of DNA and chromatin replication, and presents a hypothesis based on observations of the action of phorbol esters on lymphocytes.

Effects of tumour promoters on mouse skin are considered in the next section, and include studies of the kinetics of skin papillomas, the role of cyclic nucleotides, the relation of specific proteins in the epidermis and the information obtained from studies of the inhibition of tumour promotion by steroids. Certain of the biochemical evidence would support the view that tumour promotion may induce a dedifferentiation by modifying gene expression. The fact that the phorbol esters act at concentrations of the order to $10^{-9}$M suggests that they may act *via* specific receptors on the cell membrane, but their mode of action has yet to be established.

In the section on *in vitro* studies, a useful comparison is made of the responses of mouse skin keratinocytes, fibroblasts and carcinoma cells by Fusenig & Samsel. They find the greatest effect on the epidermal target cells, but point out that these are derived from newborn mouse epidermis, which is not stimulated *in vivo* by local applications of TPA. The conditions for the induction of ornithine and S-adenosylmethionine decarboxylases in mouse epidermal cell cultures by tumour promoters are examined, and the effect of polyamine biosynthesis on cellular control is suggested as an important aspect of the effect of tumour promoters. One point to emerge is that an anti-inflammatory steroid which inhibits both tumour promotion in mouse skin *in vivo* and promoter-stimulated DNA synthesis in mouse epidermal cells *in vitro*, actually potentiates ornithine decarboxylase activity *in vivo* and *in vitro*. This section ends with an interesting paper on an organ culture/cell culture system for studying respiratory carcinogenesis, in which epithelial-cell cultures derived from tracheal explants exposed to the carcinogen MNNG and the tumour promoter TPA were examined.

The section on the effects of tumour promoters on biological systems is in some respects an extension of the section in *in vitro* studies. The opening paper is on the role of protease inhibitors in preventing the induction of a special repair system (SOS-repair) by tumour promoters. There are also papers in which a more detailed appraisal of the membrane effects have been made, and studies of effects on lymphocytes, granulocytes and platelets, without any conclusive evidence of their applicability to the two-stage carcinogenic process.

Alternative systems for studying tumour promotion are next considered. Four papers deal with hepatocarcinogenesis, in which different stages in tumour induction are clearly identified, and methods for promoting tumour formation discussed. This is a very promising system for future work. There are also individual papers on tumour promotion in the colon, lung and bladder.

The final section on modifiers of carcinogenesis is concerned mainly with co-carcinogenesis, but does include a paper on the potential of retinoids as inhibitors of tumour formation after initiation has occurred.

There is a substantial variation in the quality of individual contributions, but this book contains a great deal of interesting information. The format is of a high standard and the provision of a subject index is a considerable help. This volume is based on a symposium sponsored by the Biology Division of Oak Ridge National Laboratory and held at Gatlinburg, Tennessee in March 1977. In the past it has been the practice to publish the proceedings of these symposia, together with points raised in discussion, as a supplement to a scientific journal. We would recommend that this practice be resumed and that the papers presented at the meeting together with the associated discussions were again made available as soon as possible. The present arrangement seems to be a compromise in which substantial reviews are included together with minor contributions, and the time interval to publication is inevitably extended.

M. Fox
A. W. Craig